# Target Localization Using Double-Sided Bistatic Range Measurements in Distributed MIMO Radar Systems

**DOI:** 10.3390/s19112524

**Published:** 2019-06-02

**Authors:** Hyuksoo Shin, Wonzoo Chung

**Affiliations:** Division of Computer and Communications Engineering, Korea University, Seoul 02841, Korea; shs727@korea.ac.kr

**Keywords:** distributed MIMO radar, target localization, double-sided bistatic range (BR)

## Abstract

We develop a novel approach improving existing target localization algorithms for distributed multiple-input multiple-output (MIMO) radars based on bistatic range measurements (BRMs). In the proposed algorithms, we estimate the target position with auxiliary parameters consisting of both the target–transmitter distances and the target–receiver distances (hence, “double-sided”) in contrast to the existing BRM methods. Furthermore, we apply the double-sided approach to multistage BRM methods. Performance improvements were demonstrated via simulations and a limited theoretical analysis was attempted for the ideal two-dimensional case.

## 1. Introduction

In distributed multiple-input multiple-output (MIMO) radar systems, target localization based on the time delays between transmitters and receivers is an attractive research topic due to its high accuracy and simplicity [1,2,3]. As target-mediated time delays are nonlinear, estimation of target location via direct analysis of these delays is difficult. Hence, several approaches seeking to linearize the relationship between the target and the time delays have been proposed [4,5,6,7,8,9,10,11,12,13,14,15]. Of these, algorithms based on bistatic range measurements (BRMs), which are the sum of target–transmitter and target–receiver distances, are introduced in [6,7,8,9,10,11,12,13,14,15].

A single stage algorithm based on BRM, introduced first in [6,7], estimates the target position with the help of auxiliary parameters (distances between the target and transmitters or distances between the target and receivers). Multistage algorithms, such as those in [8,9,10,11,12,13,14,15], further refine the target position by re-using the estimates of the first-stage BRM method and exploiting their relationships, and asymptotically attain the Cramer–Rao lower bound (CRLB) [12] assuming accurate estimates of the first stage. A recent study [15] shows that the choice of auxiliary parameters (target–transmitter side or target–receiver side) in BRM methods affects the target estimation accuracy. Therefore, a systematic approach that utilizes all available auxiliary parameters optimally is desirable.

In this paper, we propose a novel approach that utilizes both target–transmitter distances and target–receiver distances as the auxiliary parameters, to improve the mean square error (MSE) performance. Furthermore, the proposed approach can be applied to the second-stage of the multistage BRM algorithms, such as in those of [8,9,10,11,12,13,14,15]. The existing multistage algorithms can be divided into two types depending on the way of linearizing the nonlinear relations between target position and auxiliary parameters estimated in the first stage: the algorithms in [8,9,10,11,12] linearize nonlinear relationships by squaring them and the algorithms in [13,14,15] use first-order Taylor expansion to this end. We present two types of double-sided two-stage BRM algorithms by applying our approach to the most recent multistage BRM algorithms, i.e., two-stage methods using squared Taylor approximated relationships. The improved MSE performances of the proposed algorithms were demonstrated by simulations and limited theoretical analysis was attempted for an ideal two-dimensional case.

The remainder of this paper is organized as follows. We briefly review the BRM method with a distributed MIMO radar system model in Section 2. In Section 3, we develop double-sided, single- and two-stage BRM algorithms. A theoretical analysis for ideal two-dimensional target/antenna positions presented in Section 4 shows the improved MSE performance afforded by the double-sided BRM algorithm. The simulations of practical three-dimensional target/antenna positions presented in Section 5 confirm that our algorithms improve MSE performance. Our conclusions are presented in Section 6.

Table 1 lists the notations used in this paper.

## 2. System Model for BRM Based Target Localization and Problem Formulation

We consider a three-dimensional, widely separated MIMO radar system consisting of a single target located at an unknown position $x_{o}={\left[x_{o},y_{o},z_{o}\right]}^{T}$ with *M* transmitting antennae (Tx) and *N* receiving antennae (Rx) located at known positions $x_{t}\left(m\right)={\left[x_{t}\left(m\right),y_{t}\left(m\right),z_{t}\left(m\right)\right]}^{T}$,m=1,⋯,M and $x_{r}\left(n\right)={\left[x_{r}\left(n\right),y_{r}\left(n\right),z_{r}\left(n\right)\right]}^{T}$, n=1,⋯,N, respectively, and, we denote the positions of antennae as $X_{t}=\left[x_{t}\left(1\right),\cdots ,x_{t}\left(M\right)\right]$ and $X_{r}=\left[x_{r}\left(1\right),\cdots ,x_{r}\left(N\right)\right]$, together.

The bistatic range (BR) between the *m*th Tx and the *n*th Rx, denoted by $r_{mn}$, is defined as the sum of the distance from the *m*th Tx to the target, denoted by $d_{t}\left(m\right)=∥x_{o}-x_{t}\left(m\right)∥$, and the distance from the target to the *n*th Rx, denoted by $d_{r}\left(n\right)=∥x_{o}-x_{r}\left(n\right)∥$ ([16]):

(1) $$\begin{array}{c} r_{mn}=d_{t}\left(m\right)+d_{r}\left(n\right). \end{array}$$

Each BR is measured by converting the estimated time delay between a Tx and an Rx to a distance. Any BR measurement (BRM) between the *m*th Tx and the *n*th Rx, denoted by ${\hat{r}}_{mn}$, is often corrupted by measurement error, denoted by $\omega_{mn}$ and modeled as an i.i.d., zero-mean white Gaussian noise with variance $\sigma_{\omega }^{2}$ ([4]):

(2) $$\begin{array}{c} {\hat{r}}_{mn}=r_{mn}+\omega_{mn}. \end{array}$$

The goal of BRM based target localization is to estimate the target location $x_{o}$ from the BRMs ${\left\{{\hat{r}}_{mn}\right\}}_{m=1,\cdots ,M,\;n=1,\cdots ,N}$.

The BRM method in [6,7] jointly estimates the target location, $x_{o}$, and the distances from Txs to the target, denoted by $d_{t}={\left[d_{t}\left(1\right),\cdots ,d_{t}\left(M\right)\right]}^{T}$, from the BRMs, using the following linear model in the presence of noise:

(3) $$\begin{array}{c} \begin{array}{c} b_{t}=\left[1_{M\times 1}⊗X_{r}^{T}-X_{t}^{T}⊗1_{N\times 1},-R_{t}\right]{\left[x_{o}^{T},d_{t}^{T}\right]}^{T}+\varepsilon_{t}, \end{array} \end{array}$$

where

(4) $$\begin{array}{c} \begin{array}{c} b_{t}=\frac{1}{2}\left[\begin{array}{c} {∥x_{r}\left(1\right)∥}^{2}-{\hat{r}}_{11}^{2}-{∥x_{t}\left(1\right)∥}^{2}\\ \vdots \\ {∥x_{r}\left(N\right)∥}^{2}-{\hat{r}}_{MN}^{2}-{∥x_{t}\left(M\right)∥}^{2} \end{array}\right] \end{array} \end{array}$$

(5) $$\begin{array}{c} \begin{array}{c} R_{t}=\mathrm{blkdiag}\left(r_{1},\cdots ,r_{M}\right), \end{array} \end{array}$$

where $r_{m}={\left[{\hat{r}}_{m1},\cdots ,{\hat{r}}_{mN}\right]}^{T}$ and $\varepsilon_{t}$ is a vector reflecting BR measurement error

([7]).

Alternatively, the BRM equation can be constructed using the distances from the target to the Rxs, denoted by $d_{r}=[d_{r}\left(1\right),\cdots$$,d_{r}{\left(N\right)]}^{T}$, instead of the $d_{t}$ values:

(6) $$\begin{array}{c} \begin{array}{c} b_{r}=\left[X_{t}^{T}⊗1_{N\times 1}-1_{M\times 1}⊗X_{r}^{T},-R_{r}\right]{\left[x_{o}^{T},d_{r}^{T}\right]}^{T}+\varepsilon_{r}, \end{array} \end{array}$$

where

(7) $$\begin{array}{c} \begin{array}{c} b_{r}=\frac{1}{2}\left[\begin{array}{c} {∥x_{t}\left(1\right)∥}^{2}-{\hat{r}}_{11}^{2}-{∥x_{r}\left(1\right)∥}^{2}\\ \vdots \\ {∥x_{t}\left(M\right)∥}^{2}-{\hat{r}}_{MN}^{2}-{∥x_{r}\left(N\right)∥}^{2} \end{array}\right] \end{array} \end{array}$$

(8) $$\begin{array}{c} \begin{array}{c} R_{r}={\left[\mathrm{diag}\left(r_{1}\right),\cdots ,\mathrm{diag}\left(r_{M}\right)\right]}^{T}, \end{array} \end{array}$$

where $\varepsilon_{r}$ is a vector reflecting BR measurement error [7]. Note that the estimated auxiliary parameters ${\hat{d}}_{t}$ or ${\hat{d}}_{r}$ contain the target information $x_{o}$. Multistage algorithms further refine the target position by exploiting this information.

The two-stage BRM method using the squared relationships ([12]) estimates the squared target position, $x_{o}⊙x_{o}$, using ${\hat{x}}_{o}$ and ${\hat{d}}_{t}$ yielded by the first-stage BRM method based on the following linear model (which reflects the relationship between ${\left[x_{o}^{T},d_{t}^{T}\right]}^{T}$ and $x_{o}⊙x_{o}$):

(9) $$\begin{array}{c} \left[\begin{array}{c} {\hat{x}}_{o}⊙{\hat{x}}_{o}\\ {\hat{d}}_{t}⊙{\hat{d}}_{t}+2X_{t}^{T}{\hat{x}}_{o}-\left(X_{t}^{T}⊙X_{t}^{T}\right)1_{3\times 1} \end{array}\right]=\left[\begin{array}{c} I_{3}\\ 1_{M\times 3} \end{array}\right]\left(x_{o}⊙x_{o}\right)+\varepsilon_{S,t}, \end{array}$$

where $\varepsilon_{S,t}$ is the error vector due to the first-stage estimation error ([12]).

Alternatively, we obtain the following linear model reflecting the relationship between ${\left[x_{o}^{T},d_{r}^{T}\right]}^{T}$ and $x_{o}⊙x_{o}$:

(10) $$\begin{array}{c} \begin{array}{c} \left[\begin{array}{c} {\hat{x}}_{o}⊙{\hat{x}}_{o}\\ {\hat{d}}_{r}⊙{\hat{d}}_{r}+2X_{r}^{T}{\hat{x}}_{o}-\left(X_{r}^{T}⊙X_{r}^{T}\right)1_{3\times 1} \end{array}\right]=\left[\begin{array}{c} I_{3}\\ 1_{N\times 3} \end{array}\right]\left(x_{o}⊙x_{o}\right)+\varepsilon_{S,r}, \end{array} \end{array}$$

where $\varepsilon_{S,r}$ is the error vector due to the first-stage estimation error [12].

Let $\hat{x_{o}⊙x_{o}}$ denote the $x_{o}⊙x_{o}$ estimated by the linear model of (9) (or (10)); then, the refined target location, denoted by ${\hat{x}}_{o,S}$, is:

(11) $$\begin{array}{c} {\hat{x}}_{o,S}=\mathrm{sgn}\left({\hat{x}}_{o}\right)⊙\sqrt{\hat{x_{o}⊙x_{o}}}. \end{array}$$

The two-stage BRM method using Taylor approximated relationships [15] considers the first-order Taylor expansion of $d_{t}\left(m\right)$ at ${\hat{x}}_{o}$ to be

(12) $$\begin{array}{c} \begin{array}{cc} d_{t}\left(m\right)={\hat{d}}_{t}\left(m\right)-▵d_{t}\left(m\right) & =∥{\hat{x}}_{o}-▵x_{o}-x_{t}\left(m\right)∥\\ & ≃∥{\hat{x}}_{o}-x_{t}\left(m\right)∥-\frac{{\hat{x}}_{o}^{T}-x_{t}^{T}\left(m\right)}{∥{\hat{x}}_{o}-x_{t}\left(m\right)∥}▵x_{o} \end{array}\;\mathrm{for}\;m=1,\cdots ,M, \end{array}$$

where $▵x_{o},▵d_{t}\left(1\right),\cdots ,▵d_{t}\left(M\right)$ are the estimation errors at the ${\hat{x}}_{o}$. The linear model reflecting the relationships of (12) is

(13) $$\begin{array}{c} \left[\begin{array}{c} 0_{3\times 1}\\ {\hat{d}}_{t}\left(1\right)-∥{\hat{x}}_{o}-x_{t}\left(1\right)∥\\ \vdots \\ {\hat{d}}_{t}\left(M\right)-∥{\hat{x}}_{o}-x_{t}\left(M\right)∥ \end{array}\right]=\left[\begin{array}{c} -I_{3}\\ \left({\hat{x}}_{o}^{T}-x_{t}^{T}\left(1\right)\right)/∥{\hat{x}}_{o}-x_{t}\left(1\right)∥\\ \vdots \\ \left({\hat{x}}_{o}^{T}-x_{t}^{T}\left(M\right)\right)/∥{\hat{x}}_{o}-x_{t}\left(M\right)∥ \end{array}\right]▵x_{o}+\left[\begin{array}{c} ▵x_{o}\\ ▵d_{t}\left(1\right)\\ \vdots \\ ▵d_{t}\left(M\right) \end{array}\right]. \end{array}$$

Alternatively, we obtain the following linear model using $d_{r}$ instead of $d_{t}$:

(14) $$\begin{array}{c} \begin{array}{c} \left[\begin{array}{c} 0_{3\times 1}\\ {\hat{d}}_{r}\left(1\right)-∥{\hat{x}}_{o}-x_{r}\left(1\right)∥\\ \vdots \\ {\hat{d}}_{r}\left(N\right)-∥{\hat{x}}_{o}-x_{r}\left(N\right)∥ \end{array}\right]=\left[\begin{array}{c} -I_{3}\\ \left({\hat{x}}_{o}^{T}-x_{r}^{T}\left(1\right)\right)/∥{\hat{x}}_{o}-x_{r}\left(1\right)∥\\ \vdots \\ \left({\hat{x}}_{o}^{T}-x_{r}^{T}\left(N\right)\right)/∥{\hat{x}}_{o}-x_{r}\left(N\right)∥ \end{array}\right]▵x_{o}+\left[\begin{array}{c} ▵x_{o}\\ ▵d_{r}\left(1\right)\\ \vdots \\ ▵d_{r}\left(N\right) \end{array}\right]. \end{array} \end{array}$$

We make an intermediate estimation of the error $▵x_{o}$ of the first stage to refine the target position. Let ${\hat{▵x}}_{o}$ denote the $▵x_{o}$ estimated by the linear model of (13) (or (14)); then, the refined target position, denoted by ${\hat{x}}_{o,A}$, is:

(15) $$\begin{array}{c} {\hat{x}}_{o,A}={\hat{x}}_{o}-{\hat{▵x}}_{o}. \end{array}$$

In the existing single-sided BRM methods, $x_{o}$ and $d_{t}$, or $x_{o}$ and $d_{r}$, are used exclusively. As the BRMs are the sum of the $d_{t}$ and $d_{r}$ values, target estimation accuracy can be improved by simultaneously estimating $x_{o}$, $d_{t}$ and $d_{r}$ in the first stage, and by fully utilizing these values in the second stage. Thus, the goal of our paper is to develop target estimation schemes that use both the Tx- and Rx-sided linear models simultaneously.

## 3. The Double-Sided BRM Approach

### 3.1. The Double-Sided Single-Stage BRM Algorithm

The target estimation performance of the BRM algorithm depends on the choice of auxiliary parameters (the transmitter-side parameters $d_{t}$ or the receiver-side parameters $d_{r}$), as shown in [15]. Such dependency implies that the linear models in (3) and (6) cannot fully exploit the target information in BRM observations. Thus, by merging the two linear models in (3) and (6) into a single linear model and, consequently, simultaneously estimating the target, $d_{t}$ and $d_{r}$ values, we fully utilize all BR information for the target estimation.

To simultaneously estimate $x_{o}$, $d_{t}$ and $d_{r}$, we rewrite the two linear models of (3) and (6) as equivalent linear models with respect to ${\left[x_{o}^{T},d_{t}^{T},d_{r}^{T}\right]}^{T}$, by inserting $0_{MN\times N}d_{r}$ and $0_{MN\times M}d_{t}$:

(16) $$\begin{array}{c} \begin{array}{c} b_{t}=\left[1_{M\times 1}⊗X_{r}^{T}-X_{t}^{T}⊗1_{N\times 1},-R_{t},0_{MN\times N}\right]{\left[x_{o}^{T},d_{t}^{T},d_{r}^{T}\right]}^{T}+\varepsilon_{t}, \end{array} \end{array}$$

(17) $$\begin{array}{c} \begin{array}{c} b_{r}=\left[X_{t}^{T}⊗1_{N\times 1}-1_{M\times 1}⊗X_{r}^{T},0_{MN\times M},-R_{r}\right]{\left[x_{o}^{T},d_{t}^{T},d_{r}^{T}\right]}^{T}+\varepsilon_{r}. \end{array} \end{array}$$

Using the above linear equations, we construct a single linear model with respect to $[x_{o}^{T},d_{t}^{T}$$,d_{r}^{T}{]}^{T}$ as follows:

(18) $$\begin{array}{c} b=H{\left[x_{o}^{T},d_{t}^{T},d_{r}^{T}\right]}^{T}+\varepsilon , \end{array}$$

where $b={\left[b_{t}^{T},b_{r}^{T}\right]}^{T}$, $\varepsilon ={\left[\varepsilon_{t}^{T},\varepsilon_{r}^{T}\right]}^{T}$, and

(19) $$\begin{array}{c} H=\left[\begin{array}{ccc} 1_{M\times 1}⊗X_{r}^{T}-X_{t}^{T}⊗1_{N\times 1} & -R_{t} & 0_{MN\times N}\\ X_{t}^{T}⊗1_{N\times 1}-1_{M\times 1}⊗X_{r}^{T} & 0_{MN\times M} & -R_{r} \end{array}\right]. \end{array}$$

The weighted least squares (WLS) solution of (18), denoted by ${\left[{\hat{x}}_{o}^{T},{\hat{d}}_{t}^{T},{\hat{d}}_{r}^{T}\right]}^{T}$, is:

(20) $$\begin{array}{c} {\left[{\hat{x}}_{o}^{T},{\hat{d}}_{t}^{T},{\hat{d}}_{r}^{T}\right]}^{T}={\left(H^{T}WH\right)}^{-1}H^{T}Wb, \end{array}$$

where the diagonal weighting matrix W is:

(21) $$\begin{array}{c} W=\mathrm{diag}{\left(\sigma_{\omega }^{2}\left[\begin{array}{c} \left(d_{r}⊙d_{r}\right)⊗1_{M\times 1}\\ 1_{N\times 1}⊗\left(d_{t}⊙d_{t}\right) \end{array}\right]\right)}^{-1}. \end{array}$$

In practice, we apply the approximated W using estimated $d_{t}$ and $d_{r}$ via a least square (LS) approach (substituting an identity matrix for *W* in (20)) as in previous methods [6,7,8,9,10,11,12,13,14,15]. Note that, instead of error covariance matrix, Cov[ε], we use the diagonal terms of Cov[ε] for *W*, since Cov[ε] is not invertible here.

The analysis of Section 4 shows that our double-sided BRM method enhances the MSE of target location estimated by the existing BRM method by a factor of two, given ideal two-dimensional target/antenna positions. The numerical simulations presented in Section 5 show that our method affords a better MSE performance than the existing BRM method when dealing with practical target/antenna positions.

### 3.2. The Double-Sided Two-Stage BRM Algorithms

In this subsection, we develop two double-sided two-stage BRM algorithms by modifying the above single-sided two-stage BRM algorithms using the squared relationships [12] and the Taylor approximation [15] to fully utilize the parameters (${\hat{x}}_{o}$, ${\hat{d}}_{t}$, and ${\hat{d}}_{r}$) estimated by the first stage double-sided BRM algorithm.

#### 3.2.1. Proposed Double-Sided Two-Stage BRM Algorithm Using the Squared Relationships

As for the single-stage algorithm, we construct an extended linear model reflecting the relationships between $d_{t}$, $d_{r}$ and $x_{o}⊙x_{o}$ by merging the two single-sided linear models of (9) and (10) as the following:

(22) $$\begin{array}{c} \left[\begin{array}{c} {\hat{x}}_{o}⊙{\hat{x}}_{o}\\ {\hat{d}}_{t}⊙{\hat{d}}_{t}+2X_{t}^{T}{\hat{x}}_{o}-\left(X_{t}^{T}⊙X_{t}^{T}\right)1_{3\times 1}\\ {\hat{d}}_{r}⊙{\hat{d}}_{r}+2X_{r}^{T}{\hat{x}}_{o}-\left(X_{r}^{T}⊙X_{r}^{T}\right)1_{3\times 1} \end{array}\right]=\left[\begin{array}{c} I_{3}\\ 1_{M\times 3}\\ 1_{N\times 3} \end{array}\right]\left(x_{o}⊙x_{o}\right)+\varepsilon_{p}, \end{array}$$

where $\varepsilon_{p}$ is error vector due to the estimation error. The method of (22) provides an estimate of the squared target location, $x_{o}⊙x_{o}$, using all ${\left[{\hat{x}}_{o}^{T},{\hat{d}}_{t}^{T},{\hat{d}}_{r}^{T}\right]}^{T}$ given by the first-stage double-sided BRM algorithm. Denote ${\left[I_{3},1_{M\times 3}^{T},1_{N\times 3}^{T}\right]}^{T}$ as $H_{p}$; then, the WLS solution of (22), denoted by $\hat{x_{o}⊙x_{o}}$, is:

(23) $$\begin{array}{c} \hat{x_{o}⊙x_{o}}={\left(H_{p}^{T}W_{p}H_{p}\right)}^{-1}H_{p}^{T}W_{p}\left[\begin{array}{c} {\hat{x}}_{o}⊙{\hat{x}}_{o}\\ {\hat{d}}_{t}⊙{\hat{d}}_{t}+2X_{t}^{T}{\hat{x}}_{o}-\left(X_{t}^{T}⊙X_{t}^{T}\right)1_{3\times 1}\\ {\hat{d}}_{r}⊙{\hat{d}}_{r}+2X_{r}^{T}{\hat{x}}_{o}-\left(X_{r}^{T}⊙X_{r}^{T}\right)1_{3\times 1} \end{array}\right]. \end{array}$$

The weighting matrix $W_{p}$ is:

(24) $$\begin{array}{c} W_{p}={\left(T{\left(H^{T}WH\right)}^{-1}T^{T}\right)}^{-1}, \end{array}$$

where

(25) $$\begin{array}{c} T=2\left[\begin{array}{cc} \mathrm{diag}(x_{o}) & 0_{3\times (M+N)}\\ A^{T} & \mathrm{diag}({\left[d_{r}^{T},d_{r}^{T}\right]}^{T}) \end{array}\right] \end{array}$$

(26) $$\begin{array}{c} A=[X_{t},X_{r}]. \end{array}$$

The final target position estimate, denoted by ${\hat{x}}_{o,DS}$, is:

(27) $$\begin{array}{c} {\hat{x}}_{o,DS}=\mathrm{sgn}\left({\hat{x}}_{o}\right)⊙\sqrt{\hat{x_{o}⊙x_{o}}}. \end{array}$$

#### 3.2.2. Proposed Double-Sided Two-Stage BRM Algorithm Using the Taylor Approximated Relationships

To utilize all ${\left[{\hat{x}}_{o}^{T},{\hat{d}}_{t}^{T},{\hat{d}}_{r}^{T}\right]}^{T}$ values given by the first-stage double-sided BRM algorithm, we construct the following extended linear model which reflects the Taylor approximated relationships between ${\hat{d}}_{t}$, ${\hat{d}}_{r}$ and ${\hat{x}}_{o}$ by merging the linear models in (13) and (14):

(28) $$\begin{array}{c} \left[\begin{array}{c} 0_{3\times 1}\\ {\hat{d}}_{t}\left(1\right)-∥{\hat{x}}_{o}-x_{t}\left(1\right)∥\\ \vdots \\ {\hat{d}}_{t}\left(M\right)-∥{\hat{x}}_{o}-x_{t}\left(M\right)∥\\ {\hat{d}}_{r}\left(1\right)-∥{\hat{x}}_{o}-x_{r}\left(1\right)∥\\ \vdots \\ {\hat{d}}_{r}\left(N\right)-∥{\hat{x}}_{o}-x_{r}\left(N\right)∥ \end{array}\right]=\left[\begin{array}{c} -I_{3}\\ \left({\hat{x}}_{o}^{T}-x_{t}^{T}\left(1\right)\right)/∥{\hat{x}}_{o}-x_{t}\left(1\right)∥\\ \vdots \\ \left({\hat{x}}_{o}^{T}-x_{t}^{T}\left(M\right)\right)/∥{\hat{x}}_{o}-x_{t}\left(M\right)∥\\ \left({\hat{x}}_{o}^{T}-x_{r}^{T}\left(1\right)\right)/∥{\hat{x}}_{o}-x_{r}\left(1\right)∥\\ \vdots \\ \left({\hat{x}}_{o}^{T}-x_{r}^{T}\left(N\right)\right)/∥{\hat{x}}_{o}-x_{r}\left(N\right)∥ \end{array}\right]▵x_{o}+\left[\begin{array}{c} ▵x_{o}\\ ▵d_{t}\left(1\right)\\ \vdots \\ ▵d_{t}\left(M\right)\\ ▵d_{r}\left(1\right)\\ \vdots \\ ▵d_{r}\left(N\right) \end{array}\right], \end{array}$$

where $▵x_{o},▵d_{t}\left(1\right),\cdots ,▵d_{t}\left(M\right),▵d_{r}\left(1\right),\cdots ,▵d_{r}\left(N\right)$ are the estimation errors at ${\hat{x}}_{o}$. The method of (28) provides an estimate of $▵x_{o}$. Let us denote

(29) $$\begin{array}{c} H_{p}=\left[\begin{array}{c} -I_{3}\\ \left({\hat{x}}_{o}^{T}-x_{t}^{T}\left(1\right)\right)/∥{\hat{x}}_{o}-x_{t}\left(1\right)∥\\ \vdots \\ \left({\hat{x}}_{o}^{T}-x_{t}^{T}\left(M\right)\right)/∥{\hat{x}}_{o}-x_{t}\left(M\right)∥\\ \left({\hat{x}}_{o}^{T}-x_{r}^{T}\left(1\right)\right)/∥{\hat{x}}_{o}-x_{r}\left(1\right)∥\\ \vdots \\ \left({\hat{x}}_{o}^{T}-x_{r}^{T}\left(N\right)\right)/∥{\hat{x}}_{o}-x_{r}\left(N\right)∥ \end{array}\right]; \end{array}$$

then, the WLS solution of (28), denoted by ${\hat{▵x}}_{o}$, is:

(30) $$\begin{array}{c} {\hat{▵X}}_{o}={\left(H_{p}^{T}W_{p}H_{p}\right)}^{-1}H_{p}^{T}W_{p}\left[\begin{array}{c} 0_{3\times 1}\\ {\hat{d}}_{t}\left(1\right)-∥{\hat{x}}_{o}-x_{t}\left(1\right)∥\\ \vdots \\ {\hat{d}}_{t}\left(M\right)-∥{\hat{x}}_{o}-x_{t}\left(M\right)∥\\ {\hat{d}}_{r}\left(1\right)-∥{\hat{x}}_{o}-x_{r}\left(1\right)∥\\ \vdots \\ {\hat{d}}_{r}\left(N\right)-∥{\hat{x}}_{o}-x_{r}\left(N\right)∥ \end{array}\right] \end{array}$$

where the weighting matrix $W_{p}$ is

(31) $$\begin{array}{c} W_{p}={\left(H^{T}WH\right)}^{-1}. \end{array}$$

The final target position estimate, denoted by ${\hat{x}}_{o,DA}$, is:

(32) $$\begin{array}{c} {\hat{x}}_{o,DA}={\hat{x}}_{o}-{\hat{▵x}}_{o}. \end{array}$$

Unfortunately, theoretical performance analysis of (27) and (32) are virtually impossible given their complexity. However, the simulation results presented in Section 5 support the suggestion that our double-sided BRM method improves existing algorithms.

Table 2 compares the overall complexity of the double-sided algorithms to that of single-sided algorithms in terms of the number of multiplications.

The extra complexity of the double-sided algorithms is attributable principally to the larger matrix used for WLS computation. The increased computation cost scales polynomially, but is acceptable given the performance gain demonstrated by the simulations presented in Section 5.

## 4. Performance Analysis of Double-Sided BRM Method for Ideal Target/Antennae Positions

Here, we derive target estimation MSEs of our double-sided BRM method and the BRM method of Noroozi [7] when the two-dimensional target/antenna positions are ideal. Derivation of general, theoretical MSEs of target estimations is extremely complicated; the existing study in [7] assumes that the target/antenna distributions in the *x*-*y* plane are ideal. Accepting this, let the target be at (without loss of generality) $x_{o}={\left[0,0\right]}^{T}$, and let the antennae be located uniformly around the target:

(33) $$\begin{array}{c} \begin{array}{c} x_{t}\left(m\right)=d{\left[\cos\left(\theta_{0}+\frac{2\pi m}{M}\right),\sin\left(\theta_{0}+\frac{2\pi m}{M}\right)\right]}^{T},\\ x_{r}\left(n\right)=d{\left[\cos\left(\phi_{0}+\frac{2\pi n}{N}\right),\sin\left(\phi_{0}+\frac{2\pi n}{N}\right)\right]}^{T}, \end{array} \end{array}$$

where *d* is the common distance between the target and the various antennae, and $\theta_{0}$ and $\phi_{0}$ are distinct angles.

Assuming small BR errors, the error covariance matrix of the WLS estimator can be derived from [17,18]:

(34) $$\begin{array}{c} Cov\left[{\left[{\hat{x}}_{o}^{T},{\hat{d}}_{t}^{T},{\hat{d}}_{r}^{T}\right]}^{T}-{\left[x_{o}^{T},d_{t}^{T},d_{r}^{T}\right]}^{T}\right]={\left(H_{o}^{T}WH_{o}\right)}^{-1}H_{o}^{T}WCov\left[\varepsilon \right]WH_{o}{\left(H_{o}^{T}WH_{o}\right)}^{-1}, \end{array}$$

where $H_{o}$ is the noise-free version of H (derived by substituting $r_{mn}$ for ${\hat{r}}_{mn}$ in (19)). Accepting the above assumption, $d_{t}$ and $d_{r}$ simplify to $d1_{M\times 1}$$d1_{N\times 1}$, respectively, hence, the weighting matrix W of (21) and the covariance matrix of $\varepsilon ={\left[\varepsilon_{t}^{T},\varepsilon_{r}^{T}\right]}^{T}$, Cov[ε], simplify to:

(35) $$\begin{array}{c} W=1/\left(d^{2}\sigma_{\omega }^{2}\right)I_{2MN} \end{array}$$

(36) $$\begin{array}{c} Cov\left[\varepsilon \right]=d^{2}\sigma_{\omega }^{2}\left[\begin{array}{cc} I_{MN} & I_{MN}\\ I_{MN} & I_{MN} \end{array}\right]. \end{array}$$

As the antennas are uniformly located on a circle of radius *d*, the assumption further yields the following properties (the results for Rxs are the same):

(37) $$\begin{array}{c} \sum_{m=1}^{M}x_{t}\left(m\right)=\sum_{m=1}^{M}y_{t}\left(m\right)=0 \end{array}$$

(38) $$\begin{array}{c} \sum_{m=1}^{M}x_{t}^{2}\left(m\right)=\sum_{m=1}^{M}y_{t}^{2}\left(m\right)=Md^{2}/2. \end{array}$$

Using (37) and (38), each term of (34), ${\left(H_{o}^{T}WH_{o}\right)}^{-1}$ and $H_{o}^{T}WCov\left[\varepsilon \right]WH_{o}$, can be simplified as follows:

(39) $$\begin{array}{c} {\left(H_{o}^{T}WH_{o}\right)}^{-1}=\frac{\sigma_{\omega }^{2}}{MN}\left[\begin{array}{cc} I_{2} & \frac{1}{2d}A\\ \frac{1}{2d}A^{T} & B \end{array}\right] \end{array}$$

(40) $$\begin{array}{c} H_{o}^{T}WCov\left[\varepsilon \right]WH_{o}=\frac{1}{\sigma_{\omega }^{2}}\left[\begin{array}{cc} 0_{2\times 2} & 0_{2\times (M+N)}\\ 0_{(M+N)\times 2} & D \end{array}\right] \end{array}$$

where A is that of (26), and

(41) $$\begin{array}{c} D=\left[\begin{array}{cc} 4NI_{M} & 41_{M\times N}\\ 41_{N\times M} & 4MI_{N} \end{array}\right], \end{array}$$

hence,

(42) $$\begin{array}{c} \begin{array}{cc} Cov[{\left[{\hat{x}}_{o}^{T},{\hat{d}}_{t}^{T},{\hat{d}}_{r}^{T}\right]}^{T} & -{\left[x_{o}^{T},d_{t}^{T},d_{r}^{T}\right]}^{T}]\\ & =\sigma_{\omega }^{2}\left[\begin{array}{cc} \frac{1}{4d^{2}}ADA^{T} & \frac{1}{2d}ADB\\ \frac{1}{2d}BDA & BDB \end{array}\right]. \end{array} \end{array}$$

As the MSEs of the *x* and *y* components are the (1,1) and (2,2) elements of $Cov[{\left[{\hat{x}}_{o}^{T},{\hat{d}}_{t}^{T},{\hat{d}}_{r}^{T}\right]}^{T}-{\left[x_{o}^{T},d_{t}^{T},d_{r}^{T}\right]}^{T}]$, we are interested only in $\left(1/4d^{2}\right)ADA^{T}$. Using (38) once more, $\left(1/4d^{2}\right)ADA^{T}$ is:

(43) $$\begin{array}{c} \frac{1}{4d^{2}}ADA^{T}=\frac{1}{MN}I_{2}. \end{array}$$

Thus, we finally obtain

(44) $$\begin{array}{c} E\left\{{\left({\hat{x}}_{o}-x_{o}\right)}^{2}\right\}=E\left\{{\left({\hat{y}}_{o}-y_{o}\right)}^{2}\right\}=\frac{\sigma_{\omega }^{2}}{MN}. \end{array}$$

Meanwhile, under the same assumption, the MSEs of the existing BRM method in [7] are:

(45) $$\begin{array}{c} E\left\{{\left({\hat{x}}_{BRM}-x_{o}\right)}^{2}\right\}=E\left\{{\left({\hat{y}}_{BRM}-y_{o}\right)}^{2}\right\}=\frac{2\sigma_{\omega }^{2}}{MN}. \end{array}$$

A comparison of (44) and (45) shows that our method improves the MSE performance of the BRM method by a factor of two, given the assumed two-dimensional target/antenna positioning. As presented in the following section, simulations highlighted the improvements afforded by our algorithms when practical target/antenna settings were evaluated.

## 5. Numerical Simulation for Practical Target/Antennae Positions

**Figure 1:**
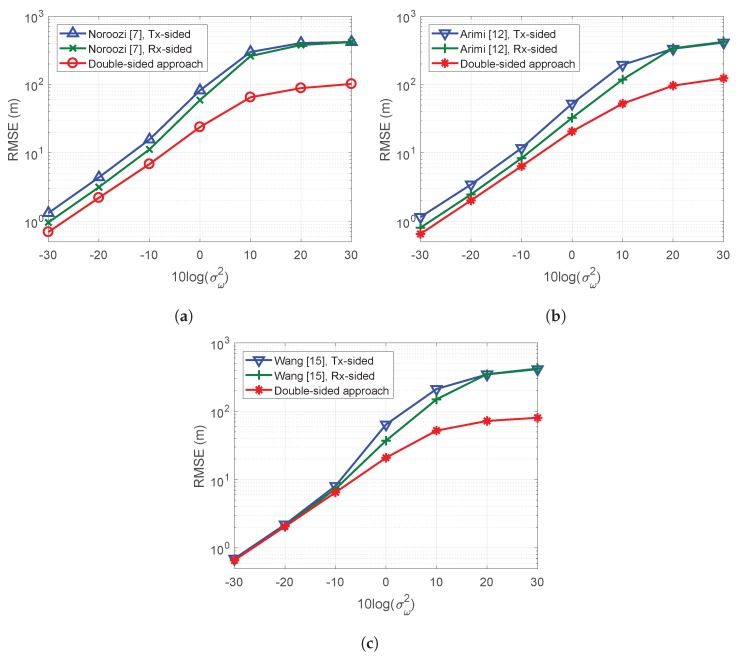
Target estimation MSE of the double-sided and single-sided algorithms with respect to noise variance: (**a**) single-stage; (**b**) two-stage using squared relations; and (**c**) two-stage using approximated relations.

Figure 1 presents the MSE performances of the proposed algorithms for the antenna positions specified in Table 3 and a target located at $x_{o}={\left[0m,0m,0m\right]}^{T}$. The results in Figure 1a show that our double-sided BRM method consistently affords better MSE performance than the single-sided BRM method of Noroozi [7], and the results in Figure 1b,c show that the double-sided two-stage BRM algorithms afford better MSE performance than the single-sided two-stage BRM methods of Amiri [12] and Wang [15].

**Figure 2:**
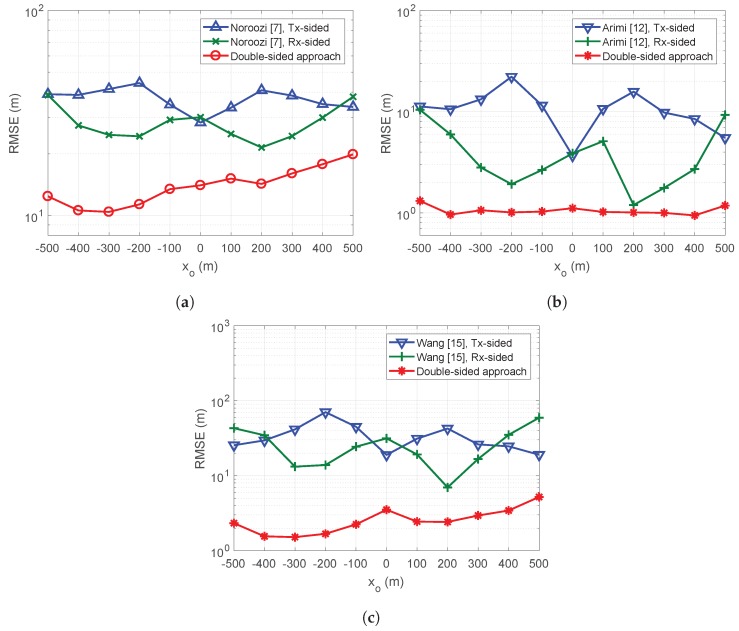
Target estimation MSE of the double-sided and single-sided algorithms with respect to the target position: (**a**) single-stage; (**b**) two-stage using squared relations; and (**c**) two-stage using approximated relations.

Figure 2 presents the MSEs of target estimations when the target moves along the *x*-axis with the *y* and *z* target positions fixed at $y_{o}=400$ m and $z_{o}=100$ m, and antennas positioned as specified in Table 4. Here, the noise variance, $\sigma_{\omega }$, was considered to be 5 m${}^{2}$. The simulations shown in Figure 2 revealed that our algorithms afforded better MSE performance than existing algorithms for all target positions tested.

## 6. Conclusions

Here, we develop a novel target localization approach improving the target estimation accuracy of existing BRM based algorithms for distributed MIMO radars. The proposed double-sided BRM method estimates target, target–transmitter, and target–receiver distances simultaneously. We also took a double-sided approach to two-stage BRM methods. The improvements afforded by the proposed algorithms were confirmed theoretically for an ideal scenario, and via numerical simulations for practical scenarios.

## Figures and Tables

**Table 1:** List of notations.

Notations	Definition
$0_{i\times j}$	i×j matrices, all elements of which are zero
$1_{i\times j}$	i×j matrices, all elements of which are unity
$I_{i}$	i×i identity matrix
diag(·)	Diagonal matrix generated from an input vector
blkdiag(·)	Block diagonal matrix generated from input vectors (or matrices)
⊗	Kronecker product
⊙	Element-wise product
sgn(·)	sign function
$\sqrt{\cdot }$	element-wise square root of the input vector

**Table 2:** Complexity table of the target localization algorithms.

Methods	Number of Multiplications
Single-sided BRM algorithm [7]	${\left(M+3\right)}^{3}+\left(2MN+2M+7\right)MN\left(M+3\right)$
Single-sided two-stage BRM algorithms ([12,15])	$\begin{array}{c} {\left(M+3\right)}^{3}+\left(2MN+2M+7\right)MN\left(M+3\right)\\ +3^{3}+3\left(M+3\right)\left(2M+13\right) \end{array}$
Double-sided BRM algorithm	${\left(M+N+3\right)}^{3}+2\left(4MN+2M+2N+7\right)MN\left(M+N+3\right)$
Double-sided two-stage BRM algorithms	$\begin{array}{c} {\left(M+N+3\right)}^{3}+2\left(4MN+2M+2N+7\right)MN\left(M+N+3\right)\\ +3^{3}+3\left(M+N+3\right)\left(2M+2N+13\right) \end{array}$

**Table 3:** Transmitters and receiver Positions (*m*).

*k*	$x_{t}\left(k\right)$	$y_{t}\left(k\right)$	$z_{t}\left(k\right)$	$x_{r}\left(k\right)$	$y_{r}\left(k\right)$	$z_{r}\left(k\right)$
1	250	300	180	−250	−300	−180
2	300	350	120	−300	−350	−120
3	300	250	160	−300	−250	−160
4	200	320	150	−200	−320	−150
5	250	200	150	−250	−200	−150
6	200	200	200	-	-	-
7	300	300	300	-	-	-

**Table 4:** Transmitters and Receiver Positions (*m*).

*k*	$x_{t}\left(k\right)$	$y_{t}\left(k\right)$	$z_{t}\left(k\right)$	$x_{r}\left(k\right)$	$y_{r}\left(k\right)$	$z_{r}\left(k\right)$
1	0	0	15	−450	−450	20
2	−300	−200	15	−450	450	30
3	−300	200	10	450	−450	40
4	−200	−300	20	450	450	10
5	−200	300	10	0	600	20
6	200	−300	10	600	0	10
7	200	300	8	−600	0	15
8	300	−200	12	0	−600	10
9	300	200	16	-	-	-
